# New Strategies for Treatment of Inflammatory Bowel Disease

**DOI:** 10.3389/fmed.2014.00003

**Published:** 2014-03-24

**Authors:** Ole Haagen Nielsen

**Affiliations:** ^1^Department of Gastroenterology, Medical Section, Herlev Hospital, University of Copenhagen, Copenhagen, Denmark

**Keywords:** biologics, Crohn’s disease, pro-inflammatory cytokines, signaling pathways, treatment, ulcerative colitis

## Abstract

The etiology of inflammatory bowel disease (IBD), of which ulcerative colitis (UC) and Crohn’s disease (CD) are the two most prevailing entities, is unknown. However, IBD is characterized by an imbalanced synthesis of pro-inflammatory mediators of the inflamed intestine, and for more than a decade tumor necrosis factor-(TNF) α has been a major target for monoclonal antibody therapy. However, TNF inhibitors are not useful for one third of all patients (i.e. “primary failures”), and further one third lose effect over time (“secondary failures”). Therefore, other strategies have in later years been developed including monoclonal antibodies targeting the interleukin (IL)-6 family of receptors (the p40 subunit of IL-12/IL-23) as well as monoclonal antibodies inhibiting adhesion molecules (the α_4_β_7_ heterodimers), which direct leukocytes to the intestinal mucosa. Recently, small molecules, which are inhibitors of Janus kinases (JAKs), hold promise with a tolerable safety profile and efficacy in UC, and the field of nanomedicine is emerging with siRNAs loaded into polyactide nanoparticles that may silence gene transcripts at sites of intestinal inflammation. Thus, drug development for IBD holds great promise, and patients as well as their treating physicians can be hopeful for the future.

Inflammatory bowel disease (IBD), of which ulcerative colitis (UC) and Crohn’s disease (CD) are the two prevailing entities, constitutes an important global public health problem with increasing incidence (1). The disease is multifactorial driven mainly by an inappropriate immune response to gut microbes in a genetically predisposed host (2). IBD occurs worldwide but its incidence and prevalence vary widely among geographic regions (1). The increased prevalence will as a consequence translate into higher health care expenditures, and patient costs for IBD, which are higher than for asthma, hypertension, and chronic obstructive pulmonary disease (3), will become increasingly relevant to the economy as a whole (4). Additionally, recent mortality data have revealed an increase in intermediate and long-term mortality among patients with IBD with even higher percentages for patients diagnosed as children or adolescents (5).

Conventional management of IBD follow a step-up strategy (6, 7), and for several years the treatment options were glucocorticoids, immunomodulators [i.e. thiopurines and methotrexate (the latter for CD only)], cyclosporine, 5-aminosalicylic acid (for UC only), and antibiotics (8, 9), but in later years there has been a landmark of discoveries and advancements in our understanding of the innate and adaptive immune responses. These discoveries have been paralleled by an exponential increase in the number of new and investigational therapeutic targets briefly mentioned in the following (10).

## TNF Inhibitors

For one and a half decade, the treatment of more than 1.3 million patients with tumor necrosis factor (TNF-α) inhibitors have generated huge amounts of safety and long-term efficacy data. This class include monoclonal antibodies of which infliximab was first on the market, followed by adalimumab, certolizumab pegol (a Fab′ fragment), and recently golimumab (Table 1) (11). One of the drawbacks attributed to biologics is, however, the loss of response caused by antibody formation and the costs associated with long-term therapy (12). Notably, around 33% fail to respond to TNF inhibitors and another third of all patients lose response over time and need to be switched to another TNF inhibitor (11). Nevertheless, prospective randomized controlled trials have demonstrated that combination therapy with thiopurines and infliximab is superior to either agent alone in both UC and CD (13, 14). Thus, combination therapy reduces anti-infliximab antibodies and approximately doubles the level of infliximab in circulation (13, 15). Altogether, these data suggest that concomitant therapy leads to optimized clinical outcomes and that the use of combination therapy in IBD is likely to increase (16). Nevertheless, it should be noticed that the underlying modes of action of the available TNF inhibitors are rather complex (17).

**Table 1:** **Novel drugs for treatment of inflammatory bowel disease**.

Structure	Drug	Route of administration	Indications	Target(s)
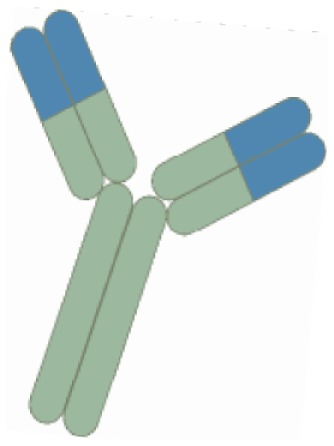	Infliximab (75% human, 25% mouse)	Intravenous	CD and UC	TNF-α
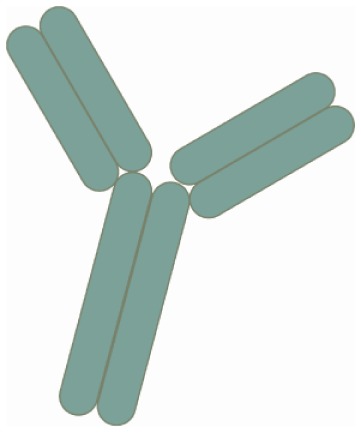	Adalimumab (100% human)	Subcutaneous injection	CD and UC	TNF-α
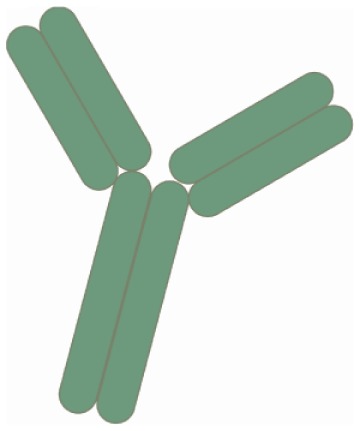	Golimumab (100% human)	Subcutaneous injection	UC	TNF-α
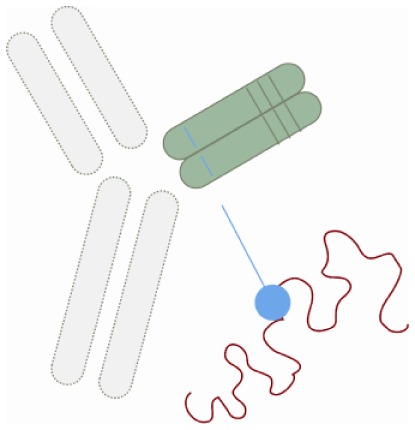	Certolizumab pegol (humanized Fab fragment)	Subcutaneous injection	CD	TNF-α
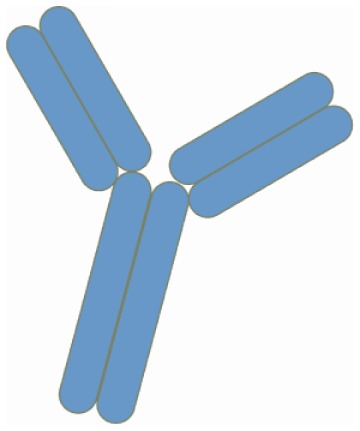	Ustekinumab (100% human)	Subcutaneous injection	CD	IL-12 and IL-23
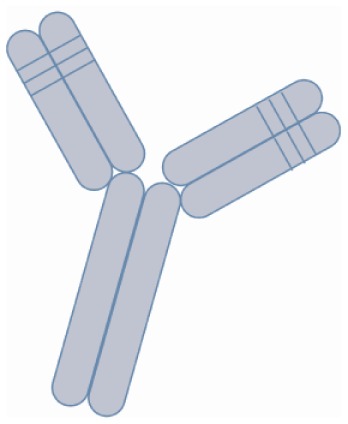	Natalizumab (humanized)	Intravenous	CD	α4β1 and α4β7
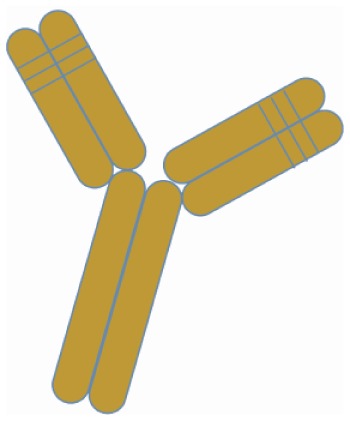	Vedolizumab (humanized)	Intravenous	CD and UC	α4β7
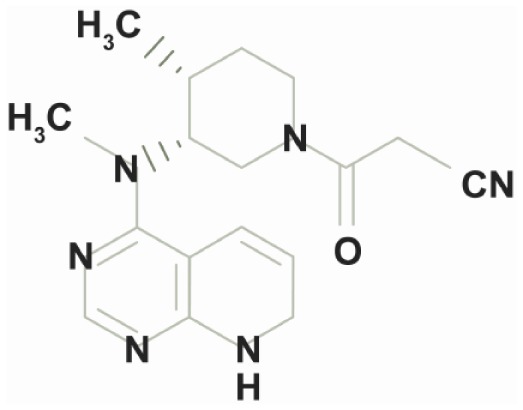	Tofacitinib (small molecule)	Oral	UC	JAK1 and JAK3
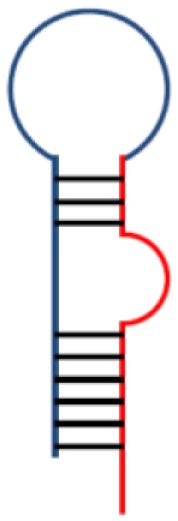	siRNA (nanomolecule)	Oral	CD and UC	siRNA targeting TNF-α transcripts

*CD, Crohn’s disease; UC, ulcerative colitis; TNF, tumor necrosis factor; IL, interleukin; JAK, Janus kinase; siRNA, small interfering RNA*.

*The double-stranded structure is degraded to single strands, and the red part is bound to target mRNA and directs it for cleavage)*.

*The structure of antibodies, small molecule or siRNA is shown (the human antibody part of TNF inhibitors is in green color)*.

There is an apparent shift in cost profile from surgery and hospitalization toward TNF inhibitor treatment, but the relatively consistent overall total costs suggest that the high cost of these biologics are partly compensated for by the reduction in surgery and hospitalization rates (18–20). Whether long-term TNF inhibitor therapy is cost effective in IBD has yet to be determined, at least from the society’s perspective. Even with early introduction to a TNF inhibitor, one in five patients with UC (21) and seven of 10 patients with CD (22) will eventually require colectomy or small bowel resections. However, careful monitoring of changes in the cost of care in IBD will ensure that timely economic decisions can be made.

Regarding the combination therapy with thiopurines and TNF inhibitors, some but not all studies show an association between this combination and adverse events, particularly non-Hodgkin lymphoma, other cancers, and opportunistic infections (23, 24). More information is needed to conclude which patients benefit from combination therapy and what is the optimal duration of treatment (11). Further, controversy exists regarding which patients with IBD require early aggressive treatment versus those who can be treated with the more conventional step-up strategy. Preliminary data for top-down therapy in CD suggest an improvement in the natural history (25). Thus, predictors of aggressive IBD may be helpful in risk stratification and to decide who will benefit from early aggressive therapy. Improved understanding of the pathogenesis of IBD as well as biomarker identification will hopefully facilitate risk stratification and identification of patients who will benefit from early top-down treatment (26, 27).

## Therapeutics Other than TNF Inhibitors

As there is a large unmet need in the therapeutic options for patients with IBD, targeting molecular pathways other than TNF-α is a recent approach in the management of IBD (Table 1) (28).

The efficacy of ustekinumab, a monoclonal antibody targeting the interleukin (IL)-6 family of receptors, namely the p40 subunit of IL-12/IL-23 (29), has been investigated in CD. While the clinical remission rates in the induction therapy did not differ significantly from placebo at week 6, the maintenance therapy with ustekinumab every 8 weeks resulted in a significantly higher clinical remission at week 22 (42%) as compared with placebo (27%, *p* = 0.03) among patients with anti-TNF resistance (30).

Adhesion molecules are also considered important targets for therapeutic intervention in IBD, and antibodies directed against cell-adhesion molecules have been developed. They suppress inflammation through inhibition of leukocyte adhesion and transmigration into inflamed tissues. The first approved adhesion inhibitor targeting the α_4_ integrin (natalizumab) was found to be effective for both induction and maintenance of CD (31, 32). However, its clinical use was hampered as cases of the fatal progressive multifocal leukoencephalopathy (PML) caused by reactivation of JC virus have been reported (33). Nonetheless, natalizumab has been approved by the FDA for CD with a “black box warning” for patients who are refractory to conventional therapy, including TNF inhibitors. However, patients must be free from concomitant immunosuppressants and screened for JC virus antibodies before this drug is administered, although the predictive value of this test for PML has been a matter of debate (33). Natalizumab-associated PML is caused by non-selective inhibition of the α_4_β_1_ heterodimers, which directs leukocytes not only to intestinal mucosa but also to the central nervous system. Therefore, a “second generation” drug class, which specifically inhibit intestinal β_7_ integrins have been developed (vedolizumab). Although no patients treated with vedolizumab so far acquired the infection, future occurrences should not be excluded, especially as few patients developed transient JCV viremia albeit without clinical or MRI findings (34). Hence, a FDA advisory panel concluded in December 2013 that the benefits of these new drugs may outweigh the potential risk of the drug causing PML.

Vedolizumab has been studied in UC and CD (35, 36) and after 52 weeks of treatment, 45% of patients with UC treated with vedolizumab were corticosteroid-free compared to 16% in the placebo arm, and 56% had mucosal healing (20% in the placebo arm) (35). In CD the effect was somewhat weaker (37 versus 22% in the placebo arm) (36). The favorable safety profile of vedolizumab and the stable response and remission rates over 1 year are true assets for this compound.

Apart from vedolizumab, other anti-β_7_ antibodies are in the pipeline including etrolizumab (specific for leukocyte β_7_ integrin and E-cadherin) (37) and PF-00547,659 specific for endothelial MAdCAM-1 (38). The latter mentioned drug has, however, not been associated with activation of JCV viremia (39).

Small molecules such as tofacitinib, a non-biologic oral inhibitor of Janus kinases (JAKs) selective for JAK1 and JAK3 (40, 41), hold promise with a tolerable safety profile and in contrast to the drugs already mentioned it can be taken orally (42). Additionally, small molecules are less expensive than antibodies. The JAK signaling pathways are responsible for signal transduction of various cytokine receptors involved in both the innate and adaptive immune compartments of IBD (43, 44). In UC tofacitinib has been very successful with a clinical response at week 8 of up to 78% compared with 42% in the placebo arm (41). However, this drug has no efficacy in CD (45).

Nanomedicine is an emerging area using nanotechnology for medical purposes. It refers to the utilization of molecules at the nanometer scale, and its potential is to turn molecular discoveries arising from systems biology into benefit for patients (46, 47). Nanomedicine allows targeted delivery of drugs to sites of interest (e.g., inflamed intestine) (48), and may in this way minimize the required dosing and side-effects (49). Thus, preclinical investigations with oral TNF-α-targeting siRNAs loaded into polyactide nanoparticles capable of localizing and silencing gene transcripts at sites of intestinal inflammation are promising (50, 51). Although nanomedicine is still in the making, the size-dependent accumulation of nanocarriers at sites of inflamed intestine can be utilized to increase clinical efficacy, diminish side-effects and open for new delivery routes for fragile (bio)molecules. Taken together, this novel principle of therapy has a huge potential for management of IBD in the future (52).

Head-to-head comparisons of the new agents mentioned with existing therapies would advance the care of our patients and enable the best strategy for the individuals to be selected.

## Conclusion

A number of important discoveries will impact the future of IBD therapy, and the use of biomarkers and imaging will increase in the years to come. Pharmacokinetics and therapeutic monitoring will be used to customize drug dosing for individual patients. Treatment end-points will evolve from symptoms to mucosal healing; the prevention of structural damage and the prevention of disability. Concomitant therapy with thiopurines and TNF inhibitors will be used more frequently. The continued discovery of novel therapeutic targets will provide rationale for a rich pipeline of future therapeutics, which will change clinical practice at centers treating IBD.

Due to the discovery of susceptibility genes, novel cell-subsets, and insights into antigen-processing and cell-signaling, we have nowadays acquired a much better understanding of the basis of IBD (10, 17). With the arrival of targeted therapy in the form of monoclonal antibodies, small molecule inhibitors, and RNA-interference-based therapy, we will be given the tools to manipulate specific inflammatory processes. By integrating these technologies and the expanding number of candidate pathways, several new medications can be expected to reach clinical trials in the coming years. This requires a continuous revealing of biological mechanisms, drug kinetics, and potential side-effects. Thus, a close collaboration between clinicians and research laboratories, other specialties as well as the pharmaceutical industry seems to be of paramount importance in creating progress in this expanding field.

Drug development for IBD holds great promise and patients as well as gastroenterologists can be hopeful for the future.

## Conflict of Interest Statement

The author declares that the research was conducted in the absence of any commercial or financial relationships that could be construed as a potential conflict of interest.
